# Effects of *Ageratina adenophora* invasion on soil multifunctionality and microbial networks of *Pinus yunnanensis* forest in the Panxi region, China

**DOI:** 10.3389/fpls.2026.1923914

**Published:** 2026-10-21

**Authors:** Qing Wei, Cheng-Gang Qiu, Jiao Tian, Jin-Rui Yang, Luo-Bing Zhou, Ting-Rui Yan, Han-Jun Yang, Yan-Jie Wang, Yun Wu, Ya-Nan Wang, Yong-Mei Liao, Jin-Song Chen

**Affiliations:** 1 College of Life Science, Sichuan Normal University, Chengdu, China; 2 State Key Laboratory of Herbage Improvement and Grassland Agro-Ecosystems, Center for Grassland Microbiome, College of Pastoral, Agriculture Science and Technology, Lanzhou University, Lanzhou, Gansu, China; 3 Key Laboratory of Southwest China Wildlife Resources Conservation, China West Normal University (Ministry of Education), Nanchong, China

**Keywords:** nitrogen-fixing species, plant invasions, soil microbial community, soil microbial networks, soil multifunctionality

## Abstract

The Panxi region of southwestern China, an important ecological barrier in the upper reaches of the Yangtze River, is severely invaded by *Ageratina adenophora*, yet the effects of this invasion for soil functioning remain poorly understood. To address this gap, we selected *Pinus yunnanensis* forest plots with slight or heavy *A. adenophora* invasion, to investigate the effects of invasion on soil multifunctionality and microbial networks. Additionally, we selected heavily invaded plots co-occurring with the nitrogen-fixing tree *Alnus cremastogyne* to investigate whether co-occurrence with nitrogen-fixing tree mitigates the negative effects of invasion on soil multifunctionality. The results of this study are as follows. First, heavy invasion by *A. adenophora* significantly reduced soil multifunctionality, and although microbial network stability increased, this functional decline was associated with the modular and functionally constrained architecture of the networks. Second, the coexistence of nitrogen-fixing tree *A. cremastogyne* significantly mitigated the invasion-related loss of soil multifunctionality. This mitigating effect was underpinned by increased microbial network complexity and connectivity. Third, random forest analyses identified microbial network complexity as the most important biotic predictor of soil multifunctionality, whereas its network complexity was strongly associated with soil nutrient availability and microbial cohesion. Overall, our findings suggest that microbial network complexity may play a more critical role than diversity or stability in maintaining soil multifunctionality following plant invasion. Our study provides critical empirical evidence for managing invasion pressure and preserving soil health in this ecologically vital region.

## 1 Introduction

Alien plant invasion severely threatens biodiversity in ecosystems worldwide (Abrahams et al., 2019; Knapp et al., 2023; Vilà et al., 2011). Their impacts can extend into belowground ecosystem through plant-soil feedback (Cheng et al., 2025; Lone et al., 2019; Song et al., 2025). The loss of plant biodiversity and the reduction in functional redundancy may drive shifts in the soil microbial community, ultimately facilitating the invasion of alien plants (Cheng et al., 2025; Fahey and Flory, 2022). More studies are still needed to comprehensively understand the relationship between alien plant invasion and belowground ecological processes.

As a comprehensive metric including nutrient cycling, carbon sequestration, and biodiversity maintenance, soil multifunctionality has been widely used to evaluate belowground ecological processes (Garland et al., 2021; Long et al., 2025). Soil microbial community composition and interspecific interactions may play a key role in regulating soil multifunctionality (He et al., 2025). With increasing functional stability, the α-diversity of soil microbes often exhibits a positive correlation with soil multifunctionality (Liu et al., 2022; Yang et al., 2023). However, global meta-analyses have shown that, the α-diversity of soil microbes responds to invasive plants in a species-specific manner (Custer and Van Diepen, 2020; Torres et al., 2021), suggesting that changes in soil microbial communities alone may be insufficient to explain changes in soil multifunctionality following alien plant invasion. This possibility is supported by accumulating evidence. For instance, invasion by *Lantana camara* reduced soil multifunctionality in a tropical dry forest by decreasing soil nutrient availability and altering soil enzyme activities (Singh et al., 2018). Similarly, invasion by the alien tree *Ailanthus altissima* significantly reduced soil ecosystem multifunctionality in riparian habitats through both direct effects on soil processes (e.g., altered soil nitrogen pools, moisture, and pH) (Constán-Nava et al., 2015). These findings collectively suggest that alien plant invasion often leads to a decline in soil multifunctionality.

Soil microbial networks, including their topology and inter-species interactions, can be helpful to detect changes in soil multifunctionality (Xiao et al., 2025; Chen et al., 2022; Kang et al., 2025). Given that high complexity in soil microbial networks is associated with greater community stability and functional redundancy, alterations in network structure may trigger the degradation of soil ecosystem functioning (Li et al., 2023). Invasion by *Spartina alterniflora* simplified the soil fungal network, characterized by reduced complexity, primarily through altering soil pH, decreasing fungal diversity, shifting community composition, and homogenizing community structure (Zhang et al., 2021). However, invasion by *Ambrosia artemisiifolia* and *Bidens pilosa* increased soil microbial network complexity through enhanced microbial diversity, accelerated microbial succession, intensified microbial associations, and activation of beneficial microbiota that increase resource availability (Zhao et al., 2024). Therefore, the effects of invasive plants on soil microbial networks may be context-dependent.

Native to Mexico and Central America, *A. adenophora* has extensively invaded forests, shrublands, and grasslands in southwestern and southern China (Wang and Wang, 2006). Invasion of *A. adenophora* has significantly altered soil nutrient cycling, including organic matter decomposition and the transformation of nitrogen and phosphorus (Yu et al., 2014; Zhao et al., 2019). Concurrently, *A. adenophora* invasion may restructure soil microbial composition, functionality, and diversity (Xiao et al., 2014; Chen et al., 2019; Balami et al., 2017). This restructuring is driven primarily by root exudates of *A. adenophora*, which suppress the abundance of native soil microorganisms while selectively recruiting specific bacteria and fungal taxa and inhibiting others, thereby reshaping the soil microbial community composition (Balami et al., 2017; Chen et al., 2019). Moreover, *A. adenophora* invasion reduced soil fungal species richness by replacing saprophytic fungi and accumulating pathogenic fungi, thereby increasing the occurrence frequency of pathogenic taxa (Balami et al., 2017; Chen et al., 2015; Xia et al., 2021). However, little is understood about the consequences of *A. adenophora* invasion for soil microbial networks.

In the Panxi region of China, *A. adenophora* has become a dominant understory invader in *Pinus yunnanensis* forests. Soil sampling was conducted in the *P. yunnanensis* plots slightly or heavily invaded by *A. adenophora*. We aimed to investigate the effects of *A. adenophora* invasion on soil multifunctionality and microbial networks in *P. yunnanensis* forests. Specifically, we tested the following hypotheses: (1) soil multifunctionality would be significantly reduced in heavily invaded plots, given that alien plant invasion often leads to a decline in soil multifunctionality; (2) heavy *A. adenophora* invasion would decrease the complexity and stability of soil microbial networks, because *A. adenophora* invasion substantially alters soil properties and reshapes microbial community composition, potentially disrupting microbial interactions and weakening network stability. In *P. yunnanensis* stands heavily invaded by *A. adenophora*, we observed the presence of the nitrogen-fixing tree *Alnus cremastogyne* as a naturally regenerating associate. Previous studies have found that mixing with nitrogen-fixing trees may increase soil fertility, and accelerates nutrient cycling and enhances soil microbial diversity and network complexity, thereby alleviating the degradation of soil multifunctionality (He et al., 2025; Zhou et al., 2026; Perakis and Pett-Ridge, 2019). There, we hypothesized that (3) the coexistence of *A. cremastogyne* with *P. yunnanensis* in heavily invaded plots would significantly increase soil multifunctionality and microbial network complexity and stability.

## 2 Materials and methods

### 2.1 Study area

The study was conducted in the Panxi region, southwestern China (**Supplementary Figure S1**). This region is distinguished by a south subtropical monsoon climate with pronounced dry-hot valley characteristics. Mean annual precipitation is approximately 830 mm and about 80% is concentrated between May and September. Mean annual temperature is approximately 20 °C. High temperature combined with intense evapotranspiration often leads to seasonal drought during spring and summer. Acrisol is the dominant soil type in the area.

### 2.2 Experimental design

In the Panxi region, southwestern China, mature *P. yunnanensis* forest has been established since the 1970s (Wang et al., 2024). Notably, *A. adenophora* has colonized these stands so extensively that its understory cover exceeds 90% in the most heavily invaded patches. The naturally regenerating nitrogen-fixing tree *A. cremastogyne* occurs as an associate within *P. yunnanensis* stands heavily invaded by *A. adenophora*. In October 2024, we identified 15 blocks in the study area. Within each block, we set three plots (20 m × 20 m) in *P. yunnanensis* forests with varying *A. adenophora* invasion intensities and vegetation composition. In each plot, we established five 1 m × 1 m quadrats along the two diagonals, recorded all understory plant species, and visually estimated the coverage of *A. adenophora*. The mean values of the five quadrats were used as the plot-level estimates. Based on field survey results, the plots were categorized into three types: (1) *P. yunnanensis* plot slightly invaded by *A. adenophora* (SIP), where the coverage of *A. adenophora* was lower than 10%, serving as a baseline reference for minimal invasion impact; (2) *P. yunnanensis* plot heavily invaded by *A. adenophora* (HIP), where the coverage of *A. adenophora* was higher than 90% and *A. adenophora* formed a dense monodominant layer with few native understory species persisting; and (3) *P. yunnanensis* plot heavily invaded by *A. adenophora*, in which *A. cremastogyne* occurs as a naturally regenerating associate (HIM). In SIP plots, the understory species was dominated by native species, including *Cyperus rotundus*, *Deyeuxia pyramidalis*, *Heteropogon contortus* and *Anaphalis busua*, along with scattered individuals of *A. adenophora* (the coverage was lower than 10%). In contrast, in HIP and HIM plots, *A. adenophora* formed a dense monodominant layer (the coverage was higher than 90%), with *Anaphalis busua* and *Artemisia stechmanniana* as the associated species. Stand characteristics of *P. yunnanensis* and *A. cremastogyne* in the study area are provided in **Supplementary Table S2** (**Supplementary Material**). To mitigate potential spatial interference within block, adjacent plots were separated by at least 100 m. In addition, adjacent blocks were separated by at least 1000 m to ensure spatial independence.

### 2.3 Soil sampling

After surface litter removal, eight soil samples (0–20 cm) were collected in a Z-shaped pattern from each plot. These samples were mixed into a composite sample and then placed into sterile plastic bags. Therefore, we collected a total of 45 soil samples. After transportation to laboratory, the soil samples were sieved (2.00 mm) to remove roots, litter and stones. Each sieved sample was split into three fractions to determine soil physicochemical properties and enzyme activities, and extract soil DNA.

### 2.4 Measurements

#### 2.4.1 Soil physicochemical properties

Soil physicochemical properties examined in this study included soil water content (SWC), soil bulk density (BD), total nitrogen (TN), total carbon (TC), total phosphorus (TP), soil organic carbon (SOC), available phosphorus (AP), alkali-hydrolysable nitrogen (AN), ammonium nitrogen (NH_4_^+^), nitrate nitrogen (NO_3_^-^), available potassium (AK), and pH. Detailed measurement protocols for these properties are provided in the Supplementary Methods (**Appendix S1**: Analysis on soil physicochemical properties).

#### 2.4.2 Soil enzyme activities

Enzyme activities examined in this study included β-glucosidase (BG), Acid phosphatase (ALP), leucine aminopeptidase (LAP), β-N-acetylglucosaminidase (NAG), Acid Protease (AcidPro), Urease (Ure), Polyphenol Oxidase (PPO), Peroxidase (POD). Enzyme activities were measured with commercial kits (Solarbio Science and Technology Co., Ltd., Beijing, China) following the manufacturer’s protocols.

#### 2.4.3 DNA extraction, PCR amplification, and sequencing

DNA was isolated from soil samples with the E.Z.N.A.^®^ Soil DNA Kit (Omega Bio-tek, Norcross, GA, USA) following the manufacturer’s instructions. Bacterial 16S rRNA gene fragments were amplified with primers 515F (5′-GTGYCAGCMGCCGCGGTAA-3′) and 806R (5′-GGACTACNVGGGTWTCTAA-3′), and fungal internal transcribed spacer (ITS) regions were amplified with primers 1737F (5′-GGAAGTAAAAGTCGTAACAAGG-3′) and 2043R (5′-GCTGCGTTCTTCATCATCGATGC-3′). Detailed protocols for PCR amplification are provided in the Supplementary Methods (**Appendix S2**: PCR amplification protocols).

Raw FASTQ sequences were processed following a standard pipeline (see **Supplementary Methods, Appendix S3**: DNA sequencing). Briefly, sequences were demultiplexed using a custom Perl script, quality-filtered with fastp (v0.19.6), and assembled with FLASH (v1.2.7). High-quality reads were clustered into OTUs at 97% similarity using UPARSE (v11.0.667). After removing chloroplast and mitochondrial sequences, the dataset was rarefied to 20,000 sequences per sample.

### 2.5 Data processing and statistical analysis

#### 2.5.1 Soil multifunctionality analysis

These twenty parameters were integrated to quantify soil multifunctionality using principal component analysis (PCA) (Long et al., 2025). Further details on soil multifunctionality calculation are provided in the **Supplementary Methods** (**Appendix S4**: Calculation on soil multifunctional index).

#### 2.5.2 Soil microbial diversity analysis

Soil microbial diversity was quantified using a composite diversity index derived from integrated species abundance tables. Specifically, three commonly used diversity metrics (such as species richness, Shannon diversity, and Pielou’s evenness) were first standardized to a 0–1 scale following Delgado-Baquerizo et al. (2020). The standardized metrics were then averaged to generate a composite index representing overall soil microbial diversity. All individual metrics exhibited significant positive associations with the composite index (**Supplementary Figure S4**), demonstrating the robustness of the integrated diversity measure.

#### 2.5.3 Soil microbial community composition analysis

To visualize compositional variation in microbial communities, we performed principal coordinate analysis (PCoA) with Bray-Curtis dissimilarities. To test the differences of soil bacterial and fungal community composition among the three plot types, we performed permutational multivariate analysis of variance (PERMANOVA; Anderson, 2001).

To quantify the relative contributions of deterministic versus stochastic processes to microbial community assembly, we applied a null model framework. To The infer the dominant ecological processes that shape bacterial and fungal community structure, the Bray–Curtis derived from Raup–Crick (RC_Bray_) and the β-nearest taxon index (βNTI) were calculated (Stegen et al., 2013).

#### 2.5.4 Microbial network analysis

To construct soil microbial networks, we employed SparCC due to its robust performance (Weiss et al., 2016). The networks were constructed using bacterial, fungal, and archaeal OTUs to characterize soil microbial interaction patterns. To reduce noise, low-abundance and low-frequency taxa were filtered out, retaining only OTUs with a total relative abundance > 0.05% and present in at least five samples (approximately one-third of all samples). We calculated SparCC correlations by taking the median of 20 iterative runs. To assess significance, we applied 200 bootstrap samples to infer pseudo-*P* values. Only correlations with |r| ≥ 0.3 and two-sided *P* < 0.05 were considered robust and included in the network construction (Amar et al., 2023; Faraci et al., 2024; Nakatsu et al., 2015). We visualized the microbial network using Gephi (version 0.10.1). To further assess the robustness of our whole-network topological properties, we have now conducted an additional 1,000-iteration bootstrap at the sample level. For each of the 1,000 iterations, we resampled the 15 plots within each treatment with replacement and re-ran the entire SparCC procedure to reconstruct a full network. Key topological properties (number of nodes and edges, average degree, modularity, and connectance) were extracted from each iteration, and their coefficients of variation (CV) were calculated to evaluate stability. Low CV values (< 5%) would indicate that our network metrics are robust to sampling variability.

To quantify properties of microbial network at the sample level, sample-specific sub-networks were extracted by retaining only taxa present in each individual sample (Wagg et al., 2019). Subsequently, topological properties at the network level were calculated for each sub-network. Network complexity was assessed by integrating six standardized topological metrics, including average degree, clustering coefficient, path length, network diameter, connectance, and relative modularity (Jiao et al., 2019). Metrics reflecting network sparsity were transformed so that higher values consistently indicated greater complexity. Network stability was assessed using robustness analysis based on changes in natural connectivity following the sequential removal of nodes in descending order of betweenness centrality (Wu et al., 2010). Network cohesion, which reflects the overall strength of microbial interactions, was quantified as a metric that integrates taxon abundances and null-model expectations derived from pairwise correlations among taxa (Herren and McMahon, 2017). The details on computational procedure are provided in **Supplementary Methods** (**Appendix S5**. Calculation on complexity, stability and cohesion of microbial network).

#### 2.5.5 Statistical analysis

Independent samples t-tests were employed to test for differences in soil multifunctionality, microbial diversity, microbial network cohesion, microbial network complexity and microbial network stability between lightly (SIP) and heavily (HIP) invaded plots of *P. yunnanensis* forest. Then, we further performed independent samples *t*-tests to test for differences in the mentioned variables between heavily invaded plots of *P. yunnanensis* forest (HIP) and heavily invaded plots of *P. yunnanensis* forest co-occurring with *A. cremastogyne* (HIM). We applied False Discovery Rate (FDR) correction using the Benjamini-Hochberg method to the P-values derived from these two independent t-tests. Statistical significance was determined at *P* < 0.05 (SPSS Inc., Chicago, IL, USA).

Random forest regression analyses were then applied to determine (1) the relative importance of abiotic and biotic factors driving soil multifunctionality, soil microbial network stability and complexity, and (2) the effects of soil nutrients on the relative abundance of keystone microbial taxa. Predictor importance was assessed using mean squared error (%IncMSE) based on 5,000 trees, with larger values indicating greater explanatory power. Permutation tests from the “*rfPermute*” package were used to assess significance of individual predictors. All random forest analyses were built with the “*randomForest*” package in free software R 4.2.0 (R Core Team, 2025), and the “*caret*” package used for model tuning and cross-validated R² estimation.

## 3 Results

### 3.1 Effects of *A. adenophora* invasion on soil multifunctionality

Soil multifunctionality was significantly lower in heavily invaded plot (HIP) than in slightly invaded plot (SIP) (**Figure 1**). This decline was primarily driven by slowed nutrient cycling, diminished soil water retention and a decreased relative abundance of symbiotrophs (**Supplementary Figure S2**). Soil multifunctionality was significantly higher in plot with nitrogen-fixing tree as associate (HIM) than in heavily invaded plot (HIP) (**Figure 1**). Soil nutrient provisioning, nutrient cycling and water retention were significantly enhanced in plot with nitrogen-fixing tree as associate (HIM) (**Supplementary Figure S2**). These observed changes were corroborated by variations in soil physicochemical properties and enzyme activities (**Supplementary Table S1**).

**Figure 1:**
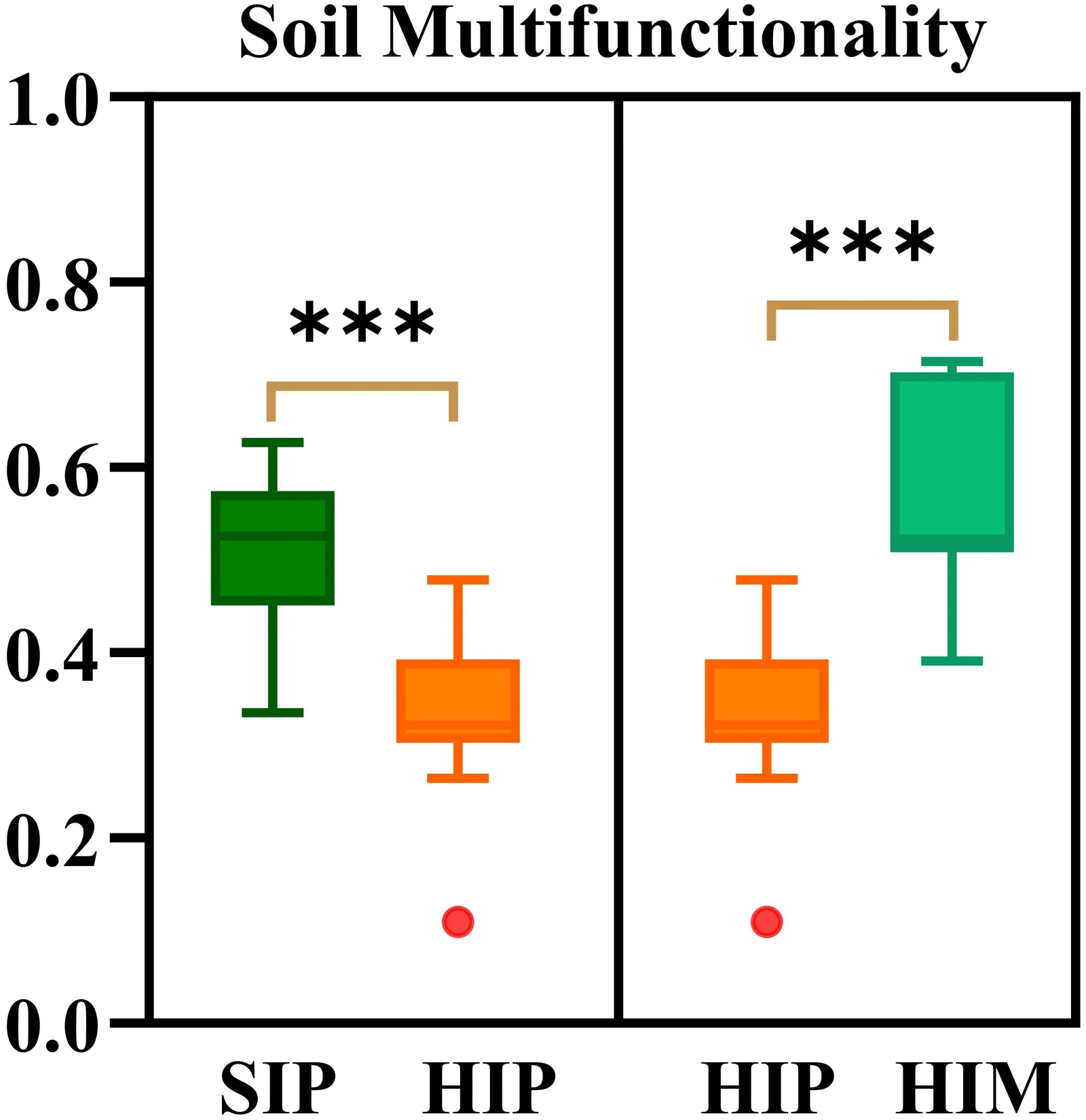
Soil multifunctionality among three types of plot: slightly invaded (understory coverage ≤ 10%; SIP) by *Ageratina adenophora*, heavily invaded (understory coverage ≥ 90%; HIP) by *A. adenophora*, heavily invaded plots (understory coverage ≥ 90%) where nitrogen-fixing tree *Alnus cremastogyne* as associate (HIM). Boxplots display medians (horizontal lines), interquartile ranges (boxes), 1.5 × interquartile ranges (whiskers), and outliers (dots). Values are presented as means ± standard error (n = 15). Significance levels are indicated as ***P < 0.001; ns, not significant.

### 3.2 Effects of *A. adenophora* invasion on soil microbial community composition and assembly

From 45 soil samples, high-throughput sequencing yielded 5534249 bacterial 16S rRNA sequences and 4570337 fungal ITS sequences. 19484 bacterial and 7255 fungal operational taxonomic units (OTUs) were generated by clustering at a 97% sequence similarity threshold. A total of 8565 OTUs were shared among the three plot types, indicating a substantial common microbial pool (**Supplementary Figure S3b**). In terms of community composition, Acidobacteriota, Proteobacteria, Chloroflexi, and Verrucomicrobiota dominated the bacterial communities in all plots, whereas Ascomycota and Basidiomycota were the predominant fungal taxa (**Supplementary Figure S3c**). Soil bacterial and fungal diversity were significantly higher in heavily invaded plot (HIP) than in slightly invaded plot (SIP) (**Figure 2a**). Soil fungal biodiversity did not differ significantly among the three plot types (**Figure 2b**). PCoA revealed clear separation in soil bacterial and fungal community composition among the three plot types, a pattern further supported by PERMANOVA results (**Figures 2c-f**).

**Figure 2:**
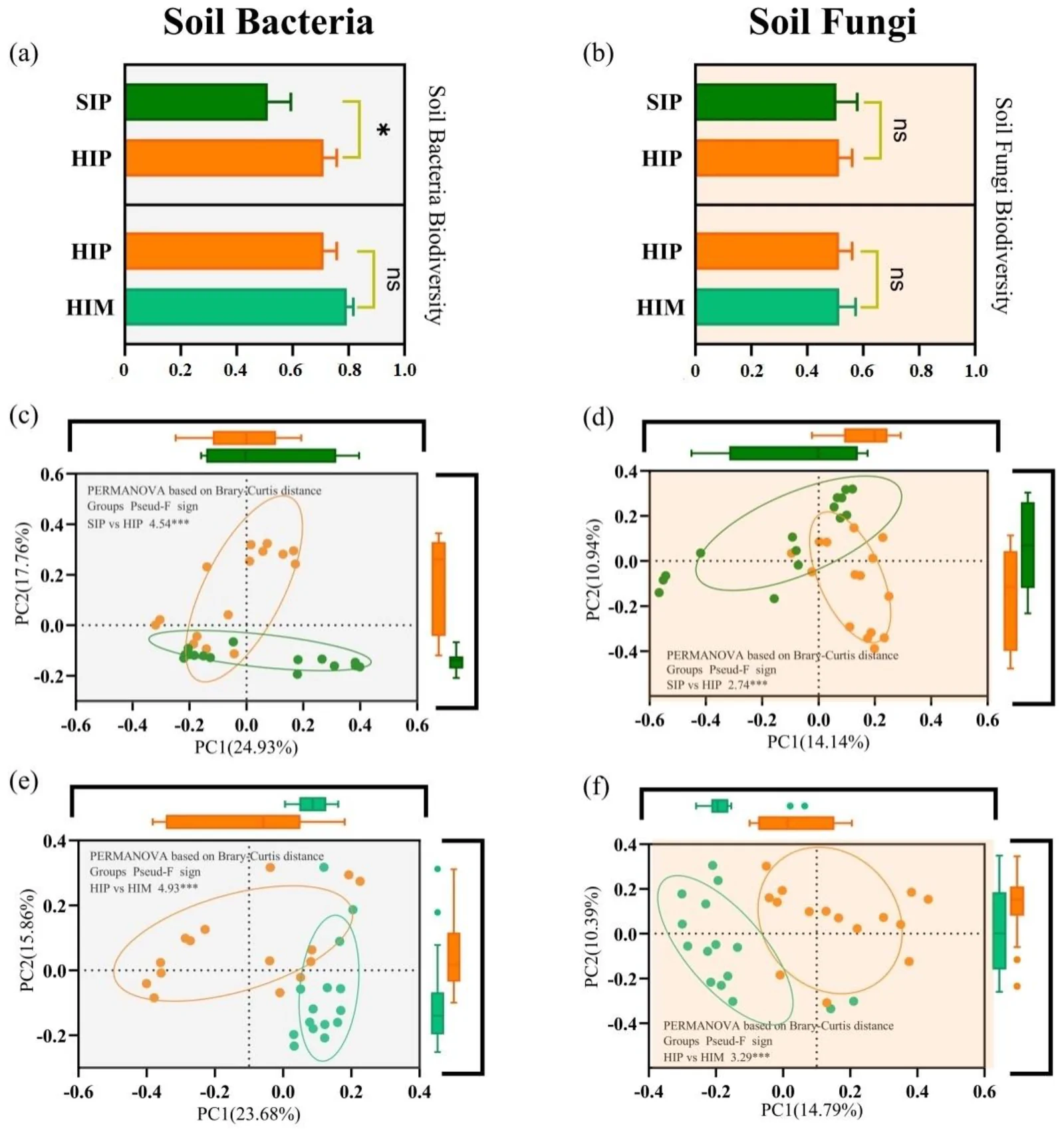
Soil microbial diversity **(a, b)** and community composition **(c–f)** among three types of plot: slightly invaded (understory coverage ≤ 10%; SIP) by *Ageratina adenophora*, heavily invaded (understory coverage ≥ 90%; HIP) by *A. adenophora*, heavily invaded plots (understory coverage ≥ 90%) where nitrogen-fixing tree *Alnus cremastogyne* as associate (HIM). Microbial community composition was tested using PERMANOVA. Values are presented as means ± standard error (n = 15). Significance levels are indicated as *P < 0.05; ns, not significant.

To understand the ecological mechanisms underlying shifts in microbial community composition, we calculated the proportional contributions of different assembly pathways. Soil bacterial community assembly was predominantly governed by heterogeneous selection across all plot types, followed by dispersal limitation, whereas homogenizing dispersal played a minor role (< 5%) (**Figures 3a, b**). In contrast, soil fungal community assembly exhibited greater stochasticity, where dispersal limitation and undominated processes collectively dominated the majority of assembly processes (**Figures 3c, d**). Overall, heterogeneous selection emerged as a more prominent assembly process of soil microbial community in heavily invaded plot (HIP) than in slightly invaded plot (SIP) or heavily invaded plot with nitrogen-fixing tree as associate (HIM).

**Figure 3:**
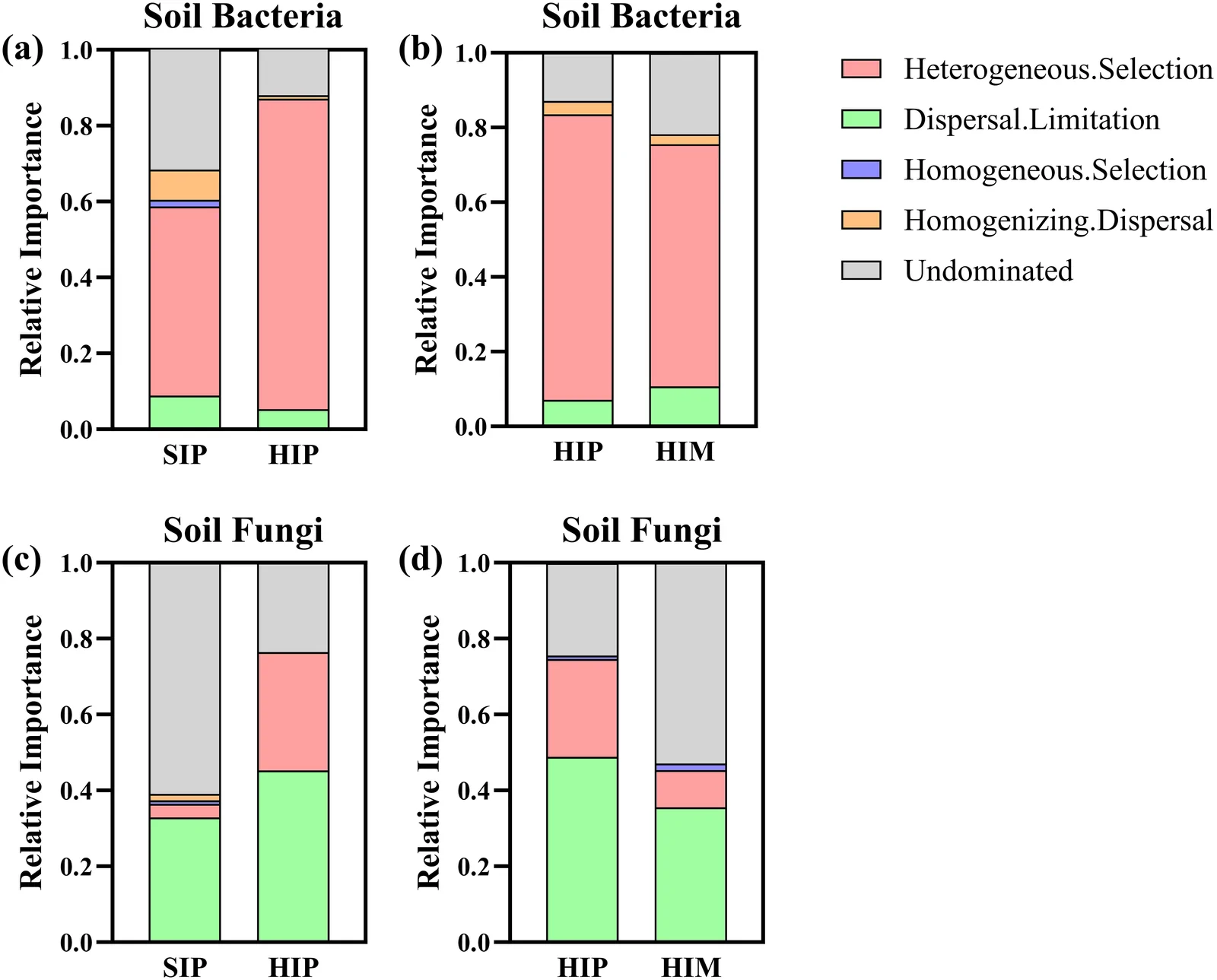
The relative importance of ecological process in soil bacteria **(a, b)** and fungi community **(c, d)** assembly among three types of plot: slightly invaded (understory coverage ≤ 10%; SIP) by *Ageratina adenophora*, heavily invaded (understory coverage ≥ 90%; HIP) by *A. adenophora*, heavily invaded plots (understory coverage ≥ 90%) where nitrogen-fixing tree *Alnus cremastogyne* as associate (HIM).

### 3.3 Effects of *A. adenophora* invasion on the complexity and stability of soil microbial network

Soil microbial network complexity was significantly lower in heavily invaded plot (HIP) than in slightly invaded (SIP; **Figures 4a, b, d, f**) or heavily invaded plot with nitrogen-fixing tree as associate (HIM; **Figures 4b, c, e, f**). Soil microbial network stability was significantly higher in heavily invaded plot (HIP) than in slightly invaded plot (SIP; **Figures 4a, b, g**). However, the network stability was significantly higher in heavily invaded plot with nitrogen-fixing tree as associate (HIM) than in heavily invaded plot (HIP) (**Figures 4b, c, g**). Total cohesion, positive cohesion, negative cohesion and ratio of negative to positive cohesion of the soil microbial network did not differ significantly between slightly invaded (SIP) and heavily invaded plots (HIP) (**Figures 4h–k**). However, total cohesion, positive cohesion, and negative cohesion of the soil microbial network were significantly higher in heavily invaded plot with nitrogen-fixing tree as associate (HIM) than in heavily invaded plot (HIP) (**Figures 4h–j**).

**Figure 4:**
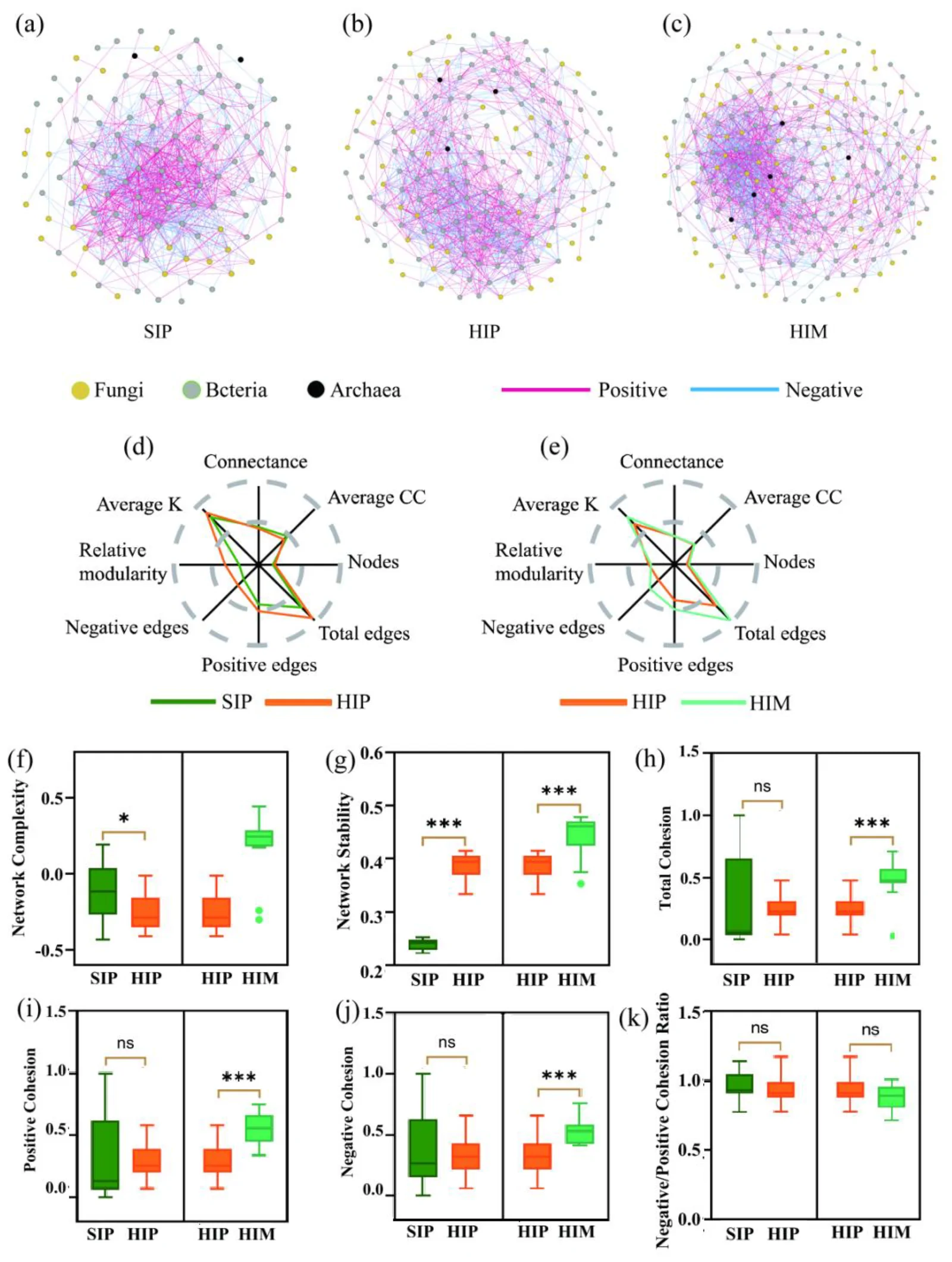
Soil microbial network based on OTU data among three types of plot: slightly invaded (understory coverage ≤ 10%; SIP) by *Ageratina adenophora*, heavily invaded (understory coverage ≥ 90%; HIP) by *A. adenophora*, heavily invaded plots (understory coverage ≥ 90%) where nitrogen-fixing tree *Alnus cremastogyne* as associate (HIM). Nodes **(a, b)** are colored according to the major groups of soil organisms, and edges are colored based on positive (red) or negative (blue) correlations. Radar plots **(d, e)** and network metrics **(f–k)** jointly illustrate difference among soil network topological properties including network complexity, network stability, total cohesion, negative cohesion, positive cohesion, and the ratio of negative to positive cohesion. Values are presented as means ± standard error (n = 15). Significance levels are indicated as *P < 0.05, **P < 0.01, ***P < 0.001; ns, not significant. Nodes **(a–c)** are colored according to the major groups of soil organisms, and edges are colored based on positive (red) or negative (blue) correlations.

### 3.4 Linking soil physicochemical and microbial properties to soil multifunctionality

Random forest analysis showed that soil bulk density (BD) was the most important abiotic predictor of soil multifunctionality, followed by total phosphorus (TP), total nitrogen (TN), and soil water content (SWC). Soil microbial network complexity was the most important biotic predictor, followed by total network cohesion (**Figure 5**).

**Figure 5:**
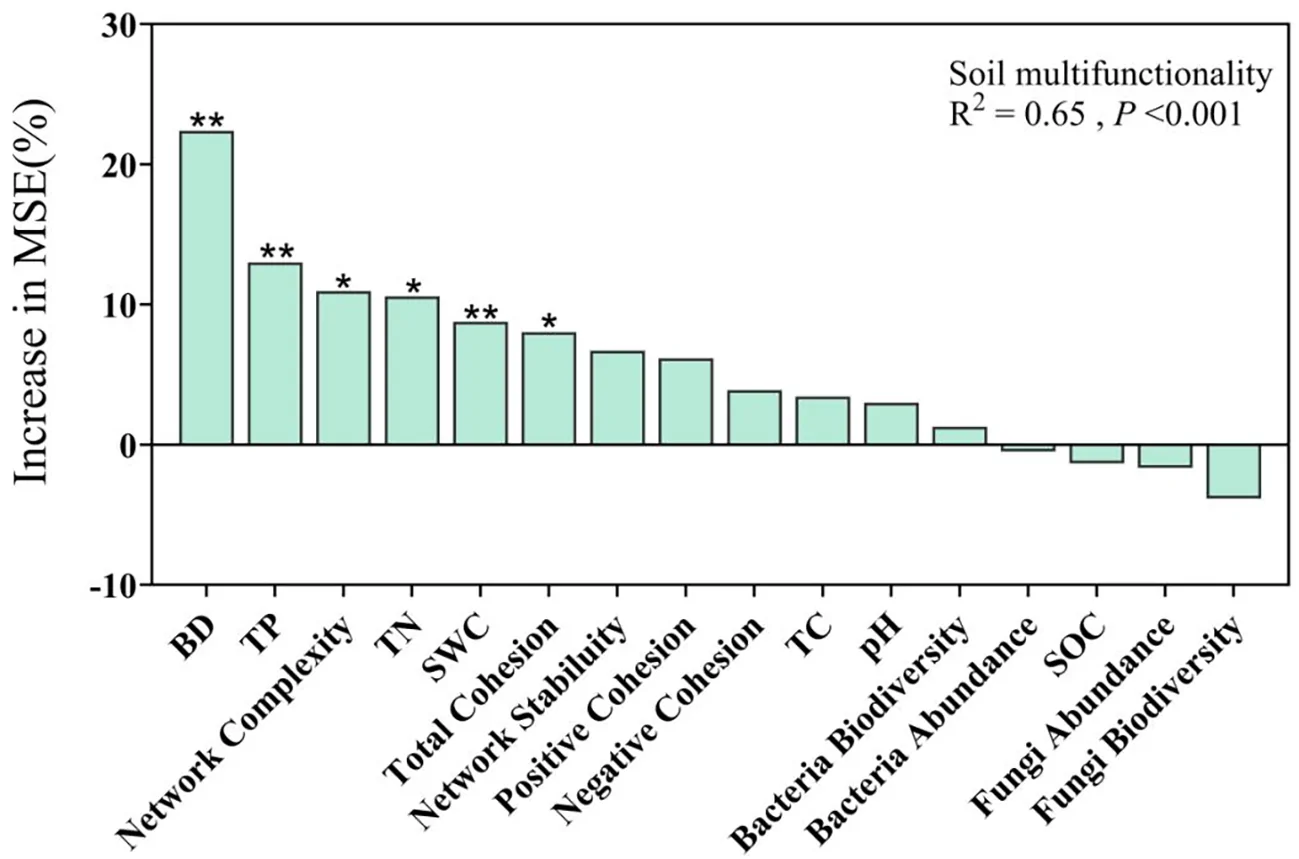
Random forest–derived importance (MSE%) of abiotic and biotic predictor for soil multifunctionality. Abiotic predictor including soil physicochemical properties except AK, AP, NH_4_^+^, and NO_3_^-^–N, whereas biotic predictor including microbial abundance and diversity, cohesion indices and network properties. Higher MSE% values indicate greater predictor importance. Significance levels are indicated as ns, not significant. No symbol indicate *P* > 0.05. *P = 0.01 –0.05, **P = 0.001 –0.01.

Random forest analysis showed that total phosphorus (TP) was the most important predictor of soil microbial network complexity, followed by total cohesion, positive cohesion, bulk density (BD), negative cohesion, alkali-hydrolyzable nitrogen (AN), total nitrogen (TN), and soil water content (SWC) (**Figure 6**). Total phosphorus (TP), total cohesion, positive cohesion, alkali-hydrolyzable nitrogen (AN), total nitrogen (TN), and soil water content (SWC) were positively associated with network complexity.

**Figure 6:**
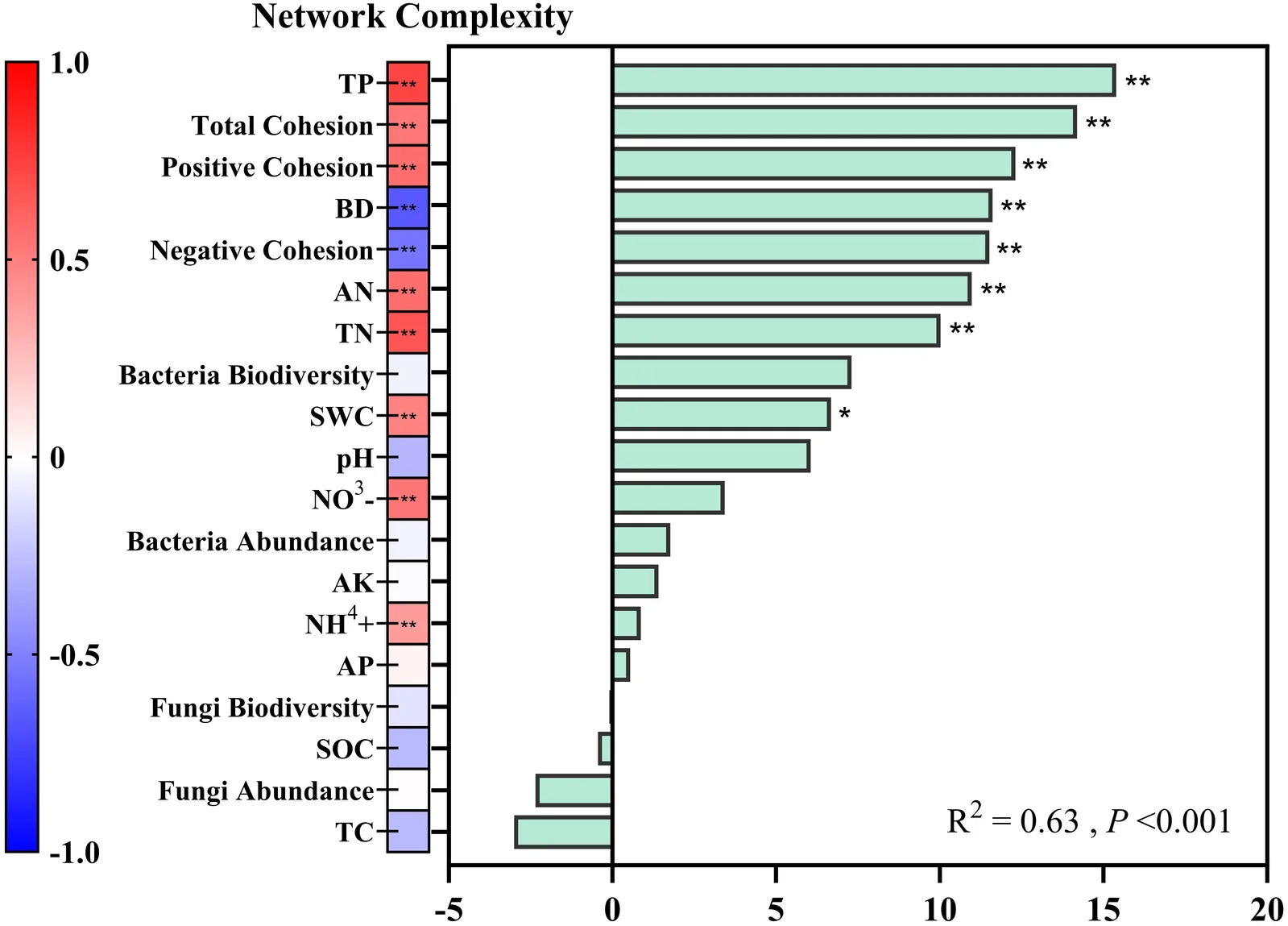
Correlation between soil abiotic or biotic factor and the complexity of soil microbial network. Abiotic factor including soil physicochemical properties, whereas biotic factor including network cohesion, soil microbial abundance and diversity. Colors indicate the direction of correlations (red for positive and blue for negative) and color intensity represents correlation strength. Random forest analysis identifies the relative importance of abiotic and biotic factors as drivers of the complexity of soil microbial network. Predictor importance is expressed as the percentage increase in mean squared error (MSE%) following permutation, calculated for each tree and averaged across 1000 trees. MSE denotes mean squared error, and higher MSE% values indicate greater predictor importance. Significance levels are indicated as ns, not significant. No symbol indicate *P* > 0.05. *P = 0.01 –0.05, **P = 0.001 –0.01.

## 4 Discussion

### 4.1 Effects of *A. adenophora* invasion on soil multifunctionality

Consistent with our first hypothesis, soil multifunctionality was significantly reduced in the *P. yunnanensis* forest heavily invaded by *A. adenophora* (**Figure 1**). As a shallow-rooted and fast-growing plant, *A. adenophora* invasion may decrease soil water retention by modifying root-mediated soil hydraulic properties (Lu et al., 2020; Tang et al., 2024). Shifts in soil structure and water retention may alter microbial populations, enzyme activities, and functional diversity, ultimately modifying soil nutrient cycling and ecosystem stability (Bogati et al., 2025). Mutualistic interaction between plant and soil microorganism was disrupted, coinciding with a decrease in the abundance of symbiotic fungi (Osayande et al., 2025). We tentatively concluded that the above-mentioned factors collectively may have contributed to the decline in soil multifunctionality after *A. adenophora* invasion (**Supplementary Figure S2**).

In the presence of the nitrogen-fixing tree *A. cremastogyne*, we observed significant increases in soil nitrogen availability (e.g., total nitrogen, ammonium nitrogen, and nitrate nitrogen) and nitrogen-cycling enzyme activities (β-N-acetylglucosaminidase, leucine aminopeptidase, and urease) (**Supplementary Table S1**). Enhanced nitrogen input and soil nitrogen availability may alleviate the nutrient limitation imposed by *A. adenophora* invasion. Accordingly, with improvement in soil nutrient availability and nutrient cycling, soil multifunctionality was significantly increased when *P. yunnanensis* and *A. cremastogyne* co-occurred. An alternative explanation is that nitrogen-fixing trees can also influence soil multifunctionality through non-nitrogen pathways. For example, their root systems may improve soil physical structure by reducing bulk density and increasing porosity, thereby enhancing soil habitat conditions (Meyer et al., 2014; Schäffer and von Wilpert, 2018). Additionally, root exudates may directly shape soil microbial community composition and functional potential, potentially contributing to the observed changes in soil multifunctionality (Zhang et al., 2022). However, we acknowledge that the current observational data do not allow a definitive partition of these mechanisms, and we suggest that future manipulative experiments (e.g., nitrogen addition vs. physical structure manipulation) are needed to disentangle their relative contributions.

Random forest analysis identified soil bulk density as the most important abiotic predictor of soil multifunctionality, underscoring the critical role of soil physical structure in regulating belowground ecological processes. Soil bulk density is a key physical property that integrates soil compaction, porosity, aeration, and water movement (Robinson et al., 2025; Rabot et al., 2018). High soil bulk density may reduce soil porosity and aeration, mechanically impedes root penetration, and restricts access to deep water and nutrients (Nawaz et al., 2013; Widmer et al., 2025), and reduce microbial abundance and suppresses the activities of key enzymes involved in carbon and nitrogen cycling (Li et al., 2002; Hu et al., 2024). In this study, the significant association between soil bulk density and reduced microbial network complexity suggests that higher soil bulk density may homogenize or fragment microbial habitats, thereby reducing the potential for intricate cross-feeding and cooperative interactions among microbial taxa. This finding is consistent with recent studies showing that alleviating soil compaction (e.g., through deep tillage) can enhance bacterial network complexity and stability (Hu et al., 2024). Together, these findings highlight soil bulk density as a key factor linking soil physical conditions to microbial network properties and soil multifunctionality.

### 4.2 Effects of *A. adenophora* invasion on assembly of soil microbial community

In this study, *A. adenophora* invasion significantly increased soil bacterial diversity (**Figure 2a**). A possible mechanism underlying this observation is that invasion-related changes in litter quality and root exudate profiles can enhance resource availability and environmental heterogeneity, thereby promoting soil bacterial diversity (Torres et al., 2021b; Wang et al., 2022). Significant effect of *A. adenophora* invasion on soil fungal diversity was not observed, suggesting that fungal communities may be less sensitive to invasion at the diversity level. Microbial community composition varied among three types of plot (**Figures 2c–f**). Specifically, *A. adenophora* invasion was associated with a higher relative abundance of Ascomycota (including saprotrophic and pathogenic fungi) and a lower relative abundance of Basidiomycota (associated with lignin decomposition) (**Supplementary Figure S3c**). These findings indicates that *A. adenophora* invasion may drive a shift in the soil fungal community from symbiosis-oriented and slow-turnover taxa towards opportunistic taxa, despite relatively stable fungal diversity (Molina et al., 2025; Voříšková and Baldrian, 2013).

Heterogeneous selection is a key deterministic process that filters microbial taxa (Zhang et al., 2025). In this study, *A. adenophora* invasion intensified the role of heterogeneous selection in the assembly of soil bacterial and fungal communities (**Figures 3a, b**). A possible explanation is that changes in nutrient availability and root exudate profiles associated with *A. adenophora* invasion promoted selective filtering of the soil microbial community (Anthony et al., 2017; Nunez-Mir and McCary, 2024). In contrast to the heavily invaded plot (HIP), a shift in community assembly from deterministic to stochastic processes was detected in plot with nitrogen-fixing tree *A. cremastogyne* as associate (HIM). This shift coincided with an increased relative abundance of Chloroflexi and a decreased abundance of Acidobacteriota (**Figure 3b**; **Supplementary Figure S3b**), suggesting that co-occurrence of nitrogen-fixing species altered microbial habitat conditions and alleviated environmental constraints on community assembly (Yuan et al., 2023; Wang et al., 2025).

### 4.3 Effects of *A. adenophora* invasion on soil microbial network

Invasion by *A. adenophora* did not significantly alter total cohesion, positive cohesion, negative cohesion, or the ratio of negative to positive cohesion of the soil microbial network (**Figures 4h-k**). These results implied that the overall strength of microbial associations was relatively resistant to *A. adenophora* invasion, despite pronounced shifts in microbial diversity, community composition, and assembly processes. Nevertheless, *A. adenophora* invasion reduced soil microbial network complexity while increasing network stability (**Figures 4f, g**). Similar patterns have been reported in previous studies, where simplified but highly modular microbial networks exhibited greater resistance to targeted disturbances (Zhang et al., 2023). In the study, the microbial network exhibited a more modular and less complex architecture (**Figure 4f**; **Supplementary Figure S5f**) in invaded plots, potentially enhancing network stability. When *P. yunnanensis* and *A. cremastogyne* co-occurred (HIM), total cohesion, positive cohesion, and negative cohesion of soil microbial network significantly increased, which in turn was linked to enhanced network complexity and stability (**Figures 4f, g**). However, the ratio of negative to positive cohesion significantly was not different between HIP and HIM (**Figure 4k**), indicating that the relative balance between positive and negative associations remained stable despite the increase in overall cohesion. These results supported our third prediction that the nitrogen-fixing tree *A. cremastogyne* enhanced the complexity and stability of soil microbial networks in the *P. Yunnanensis* forest as well as increase of soil multifunctionality. Furthermore, this shift toward a cooperation-dominated association within the soil microbial network may reflect weakened competitive interactions (Du et al., 2025). Random forest analysis further identified total cohesion as one of the most important predictors of microbial network complexity (**Figure 6**). With the positive association of total phosphorus, total nitrogen, alkali-hydrolyzable nitrogen, and soil moisture with network complexity, enhanced soil resource availability following co-occurrence of *A. cremastogyne* likely contributed to stronger microbial associations and promoted the development of more complex microbial networks.

### 4.4 Limitation

It should be noted that our study employed a space-for-time substitution design, comparing plots with different invasion intensities at a single time point to assess their effects on soil multifunctionality. Although this approach has been widely used in invasion ecology to evaluate the impacts of alien species on ecosystem structure and functioning (Thomaz et al., 2012), it cannot entirely exclude the potential interference of confounding environmental factors (such as soil parent material, microtopography, and land-use history) on the observed results. Future research should integrate long-term field monitoring with manipulative experiments (e.g., removal or addition of *A. adenophora* and N-fixing tree species) to more rigorously establish the causal relationships among *A. adenophora* invasion, microbial network complexity, and soil multifunctionality, and to further clarify the persistence of the mitigating effects of *A. cremastogyne*.

## 5 Conclusions

As our knowledge, our experiment is unique study to explore influences of *A. adenophora* invasion on soil multifunctionality and microbial networks. Invasion by *A. adenophora* significantly reduced soil multifunctionality and altered soil microbial community assembly in *P. yunnanensis* forest. When *P. yunnanensis* and nitrogen-fixing tree *A. cremastogyne* co-occurred, the degradation of soil multifunctionality associated with *A. adenophora* invasion was significantly alleviated. Meanwhile, the complexity and connectivity of soil microbial network were also significantly enhanced. Random forest analyses further revealed microbial network complexity to be the primary biological determinant of soil multifunctionality beyond abiotic drivers. Notably, microbial network complexity was tightly linked to both soil resource availability and microbial cohesion. It is suggested that microbial network complexity may play more important role than microbial diversity or network stability in maintaining soil multifunctionality after plant invasion. In addition, co-occurrence of nitrogen-fixing tree may serve as a nature-based solution to ameliorate the detrimental impact of *A. adenophora* invasion on soil functioning of *P. yunnanensis* forest in the Panxi region, China. Given that this study focused solely on the invasive plant *A. adenophora*, further research is needed to generalize these findings.

## Data Availability

The raw sequencing data presented in this study are deposited in the CNCB-NGDC Bioproject repository (https://ngdc.cncb.ac.cn/bioproject), accession number PRJCA076806.

## References

[B1] Abrahams B. Sitas N. Esler K. J. (2019). Exploring the dynamics of research collaborations by mapping social networks in invasion science. J. Environ. Manage. 229, 27–37. doi: 10.1016/j.jenvman.2018.06.051 29934131

[B2] Amar Y. Rogner D. Silva R. L. Foesel B. U. Ud-Dean M. Lagkouvardos I. . (2023). Darier’s disease exhibits a unique cutaneous microbial dysbiosis associated with inflammation and body malodour. Microbiome 11, 162. doi: 10.1186/s40168-023-01587-x PMC10369845 37496039

[B3] Anderson M. J. (2001). A new method for non‐parametric multivariate analysis of variance. Austral Ecol. 26, 32–46. doi: 10.1111/j.1442-9993.2001.01070.pp.x40046247

[B4] Anthony M. A. Frey S. D. Stinson K. A. (2017). Fungal community homogenization, shift in dominant trophic guild, and appearance of novel taxa with biotic invasion. Ecosphere 8, e01951. doi: 10.1002/ecs2.195142587375

[B5] Balami S. Thapa L. B. Jha S. K. (2017). Effect of invasive Ageratina adenophora on species richness and composition of saprotrophic and pathogenic soil fungi. Biotropia 24, 212–219. doi: 10.2323/jgam.2016.08.002

[B6] Bogati K. A. Sewerniak P. Walczak M. (2025). Unraveling the effect of soil moisture on microbial diversity and enzymatic activity in agricultural soils. Microorganisms 13, 1245. doi: 10.3390/microorganisms13061245 40572134 PMC12195373

[B7] Chen L. Fang K. Zhou J. Yang Z. P. Dong X. F. Dai G. H. . (2019). Enrichment of soil rare bacteria in root by an invasive plant *Ageratina adenophora*. Sci. Total Environ. 683, 202–209. doi: 10.1016/j.scitotenv.2019.05.220 31132698

[B8] Chen X. Liu Y. Liu H. Wang H. Yang D. Huangfu C. (2015). Impacts of four invasive asteraceae on soil physico-chemical properties and AM fungi community. Am. J. Plant Sci. 6, 2734–2743. doi: 10.4236/ajps.2015.617274

[B9] Chen W. Q. Wang J. Y. Chen X. Meng Z. X. Xu R. Duoji D. Z. . (2022). Soil microbial network complexity predicts ecosystem function along elevation gradients on the Tibetan Plateau. Soil Biol. Biochem. 172, 108766. doi: 10.1016/j.soilbio.2022.108766

[B10] Cheng C. Song W. Liu Z. K. Li B. van Kleunen M. Wu J. H. (2025). Intraspecific diversity mitigates the negative soil‐legacy impacts of an invasive plant. New Phytol. 245, 1277–1287. doi: 10.1111/nph.20261 39526440

[B11] Constán-Nava S. Soliveres S. Torices R. Serra L. Bonet A. (2015). Direct and indirect effects of invasion by the alien tree *Ailanthus altissima* on riparian plant communities and ecosystem multifunctionality. Biol. Invasions 17, 1095–1108. doi: 10.1007/s10530-014-0780-4

[B12] Custer G. F. Van Diepen L. T. A. (2020). Plant invasion has limited impact on soil microbial α-diversity: A meta-analysis. Diversity 12, 112. doi: 10.3390/d1203011230654563

[B13] Du Y. Yang Y. Wu S. N. Gao X. X. He X. Q. Dong S. K. (2025). Core microbes regulate plant-soil resilience by maintaining network resilience during long-term restoration of alpine grasslands. Nat. Commun. 16, 3116. doi: 10.1038/s41467-025-58080-2 PMC11961630 40169576

[B14] Fahey C. Flory S. L. (2022). Soil microbes alter competition between native and invasive plants. J. Ecol. 110, 404–414. doi: 10.1111/1365-2745.1380740046247

[B15] Faraci M. Bonaretti C. Dell’Orso G. Pierri F. Giardino S. Angiero F. . (2024). Association between oral and fecal microbiome dysbiosis and treatment complications in pediatric patients undergoing allogeneic hematopoietic stem cell transplantation. Sci. Rep. 14, 6708. doi: 10.1038/s41598-024-55690-6 PMC10954761 38509104

[B16] Garland G. Banerjee S. Edlinger A. Oliveira E. M. Herzog C. Wittwer R. . (2021). A closer look at the functions behind ecosystem multifunctionality: A review. J. Ecol. 109, 600–613. doi: 10.1111/1365-2745.13511

[B17] He Y. Q. Wen Y. G. Li K. X. Ye S. M. Zhang H. He F. . (2025). Responses of soil multifunctionality, microbial diversity, and network complexity to tree species mixing in *Eucalyptus* plantations. Ind. Crops Prod. 225, 120575. doi: 10.1016/j.indcrop.2025.120575

[B18] Herren C. M. McMahon K. D. (2017). Cohesion: a method for quantifying the connectivity of microbial communities. ISME J. 11, 2426–2438. doi: 10.1038/ismej.2017.91 28731477 PMC5649174

[B19] Hu R. W. Zheng B. F. Liu Y. J. Peng S. G. Gong J. Li J. H. . (2024). Deep tillage enhances the spatial homogenization of bacterial communities by reducing deep soil compaction. Soil Tillage Res. 239, 106062. doi: 10.1016/j.still.2024.106062

[B20] Jiao S. Xu Y. Q. Zhang J. Hao X. Lu Y. H. (2019). Core microbiota in agricultural soils and their potential associations with nutrient cycling. mSystems 4, e00313-18. doi: 10.1128/mSystems.00313-18 PMC6435817 30944882

[B21] Kang Y. L. Shen L. J. Li C. F. Huang Y. Chen L. D. (2025). Increased soil multifunctionality is determined by altered bacterial–fungal–protistan compositions and co‐occurrence network complexity during vegetation restoration in a Karst region, southwest China. J. Appl. Ecol. 62, 1216–1228. doi: 10.1111/1365-2664.70028

[B22] Knapp L. S. P. Coyle D. R. Dey D. C. Fraser J. S. Hutchinson T. Jenkins M. A. . (2023). Invasive plant management in eastern North American forests: A systematic review. For. Ecol. Manage. 550, 121517. doi: 10.1016/j.foreco.2023.121517

[B23] Li J. Huang X. B. Li S. F. Tang R. Su J. R. (2023). Microbial network complexity and diversity together drive the soil ecosystem multifunctionality of forests during different woodland use intensity in dry and wet season. For. Ecol. Manage. 542, 121086. doi: 10.1016/j.foreco.2023.121086

[B24] Li C. H. Ma B. L. Zhang T. Q. (2002). Soil bulk density effects on soil microbial populations and enzyme activities during the growth of maize (*Zea mays* L.) planted in large pots under field exposure. Can. J. Soil Sci. 82, 147–154. doi: 10.4141/S01-026

[B25] Liu M. H. Feng F. J. Cai T. J. Tang S. J. (2022). Fungal community diversity dominates soil multifunctionality in freeze-thaw events. Catena 214, 106241. doi: 10.1016/j.catena.2022.106241

[B26] Lone P. A. Dar J. A. Subashree K. Raha D. Pandey P. K. Ray T. . (2019). Impact of plant invasion on physical, chemical and biological aspects of ecosystems: A review. Trop. Plant Res. 3, 528–544. doi: 10.22271/tpr.2019.v6.i3.067

[B27] Long X. W. Li J. N. Liao X. H. Wang J. C. Zhang W. Wang K. L. . (2025). Stable soil biota network enhances soil multifunctionality in agroecosystems. Global Change Biol. 31, e70041. doi: 10.1111/gcb.70041 39840664

[B28] Lu J. R. Zhang Q. Werner A. D. Li Y. L. Jiang S. Y. Tan Z. Q. . (2020). Root-induced changes of soil hydraulic properties – A review. J. Hydrol. 589, 125203. doi: 10.1016/j.jhydrol.2020.125203

[B29] Meyer C. Lüscher P. Schulin R. (2014). Recovery of forest soil from compaction in skid tracks planted with black alder (*Alnus glutinosa* (L.) Gaertn.). Soil Tillage Res. 143, 83–92. doi: 10.1016/j.still.2014.05.006

[B30] Molina L. Rajchenberg M. Pildain M. B. Aime M. C. (2025). Seasonal turnover and functional structure of the foliar mycobiota in a gondwanan temperate forest keystone tree. J. Fungi 11, 795. doi: 10.3390/jof11110795 PMC12653127 41295176

[B31] Nakatsu G. Li X. C. Zhou H. K. Sheng J. Q. Wong S. H. Wu W. K. K. . (2015). Gut mucosal microbiome across stages of colorectal carcinogenesis. Nat. Commun. 6, 8727. doi: 10.1038/ncomms9727 PMC4640069 26515465

[B32] Nawaz M. F. Bourrié G. Trolard F. (2013). Soil compaction impact and modelling. A review. Agron. Sustain. Dev. 33, 291–309. doi: 10.1007/s13593-011-0071-830311153

[B33] Nunez-Mir G. C. McCary M. A. (2024). Invasive plants and their root traits are linked to the homogenization of soil microbial communities across the United States. Proc. Natl. Acad. Sci. U.S.A. 121, e2418632121. doi: 10.1073/pnas.2418632121 39446386 PMC11536171

[B34] Osayande I. S. Han X. Tsuda K. (2025). Dynamic shifts in plant-microbe relationships. Plant Biotechnol. 42, 271–277. doi: 10.5511/plantbiotechnology.25.0428a 41181069 PMC12573601

[B35] Perakis S. S. Pett-Ridge J. C. (2019). Nitrogen-fixing red alder trees tap rock-derived nutrients. Proc. Natl. Acad. Sci. U.S.A. 116, 5009–5014. doi: 10.1073/pnas.1814782116 30804181 PMC6421423

[B36] Rabot E. Wiesmeier M. Schlüter S. Vogel H. J. (2018). Soil structure as an indicator of soil functions: A review. Geoderma 314, 122–137. doi: 10.1016/j.geoderma.2017.11.009

[B37] R Core Team (2025). R: A Language and Environment for Statistical Computing (Austria: R Foundation for Statistical Computing Vienna).

[B38] Robinson D. A. Friedman S. P. Thomas A. Hirmas D. Sullivan P. L. Nemes A. (2025). Soil bulk density and porosity connecting macro- and micro-scales through geometry. Earth Sci. Rev. 268, 105173. doi: 10.1016/j.earscirev.2025.105173

[B39] Schäffer J. von Wilpert K. (2018). Planting of alder trees at the edge of skid trails helps to stabilize forest topsoil structure against damage caused by heavy forestry machines. Soil Tillage Res. 184, 51–62. doi: 10.1016/j.still.2018.12.01342574925

[B40] Singh A. K. Rai A. Banyal R. Chauhan P. S. Singh N. (2018). Plant community regulates soil multifunctionality in a tropical dry forest. Ecol. Indic. 95, 953–963. doi: 10.1016/j.ecolind.2018.08.030

[B41] Song W. Cheng C. Liu Z. He S. M. Xue S. G. Chen X. . (2025). Invasion legacy weakens the positive effect of plant genotype diversity on soil multifunctionality. Plant Soil 515, 1961–1974. doi: 10.1007/s11104-025-07697-9

[B42] Stegen J. C. Lin X. Fredrickson J. K. Chen X. Y. Kennedy D. W. Murray C. J. . (2013). Quantifying community assembly processes and identifying features that impose them. ISME J. 7, 2069–2079. doi: 10.1038/ismej.2013.93 PMC3806266 23739053

[B43] Tang Y. Y. Pan H. P. Zhang T. Cao L. X. Wang Y. (2024). The dynamics of soil macropores and hydraulic conductivity as influenced by the fibrous and tap root systems. Agriculture 14, 1676. doi: 10.3390/agriculture14101676

[B44] Thomaz S. M. Agostinho A. A. Gomes L. C. Silveira M. J. Rejmánek M. Aslan C. E. . (2012). Using space-for-time substitution and time sequence approaches in invasion ecology. Freshw. Biol. 57, 2401–2410. doi: 10.1111/fwb.12005

[B45] Torres N. Herrera I. Fajardo L. Bustamante R. O. (2021). Meta-analysis of the impact of plant invasions on soil microbial communities. BMC Ecol. Evol. 21, 172. doi: 10.1186/s12862-021-01899-2 PMC8425116 34496752

[B46] Vilà M. Espinar J. L. Hejda M. Hulme P. E. Jarošík V. Maron J. L. . (2011). Ecological impacts of invasive alien plants: a meta-analysis of their effects on species, communities and ecosystems. Ecol. Lett. 14, 702–708. doi: 10.1111/j.1461-0248.2011.01628.x 21592274

[B47] Voříšková J. Baldrian P. (2013). Fungal community on decomposing leaf litter undergoes rapid successional changes. ISME J. 7, 477–486. doi: 10.1038/ismej.2012.116 23051693 PMC3578564

[B48] Wagg C. Schlaeppi K. Banerjee S. Kuramae E. E. van der Heijden M. G. A. (2019). Fungal-bacterial diversity and microbiome complexity predict ecosystem functioning. Nat. Commun. 10, 4841. doi: 10.1038/s41467-019-12798-y PMC6813331 31649246

[B49] Wang R. Wang Y. Z. (2006). Invasion dynamics and potential spread of the invasive alien plant species *Ageratina adenophora* (Asteraceae) in China. Divers. Distrib. 12, 397–408. doi: 10.1111/j.1366-9516.2006.00250.x40046247

[B50] Wang X. J. Wang J. Chen J. Bezemer T. M. Song Z. L. Wanek W. . (2025). Environmental stresses constrain soil microbial community functions by regulating deterministic assembly and niche width. Mol. Ecol. 34, e70096. doi: 10.1111/mec.70096 40927879

[B51] Wang X. Y. Wang X. Wang W. Wang J. Yu F. H. (2022). Effects of invasive plant diversity on soil microbial communities. Diversity 14, 992. doi: 10.3390/d14110992

[B52] Wang G. Y. Zhang X. J. Yannelli F. Li J. J. Shi S. L. Zhang T. B. . (2024). The impact of species phylogenetic relatedness on invasion varies distinctly along resource versus non-resource environmental gradients. J. Appl. Ecol. 61, 869–883. doi: 10.1111/1365-2664.14607

[B53] Weiss S. Van Treuren W. Lozupone C. Faust K. Friedman J. Deng Y. . (2016). Correlation detection strategies in microbial data sets vary widely in sensitivity and precision. ISME J. 10, 1669–1681. doi: 10.1038/ismej.2015.235 PMC4918442 26905627

[B54] Widmer A. Johannes A. Fontana M. Sommer M. Elfouki S. Bragazza L. . (2025). Short-term impact of mechanical loosening on physical soil properties in a severely compacted subsoil. Geoderma 464, 117611. doi: 10.1016/j.geoderma.2025.117611

[B55] Wu J. Barahona M. Tan Y. J. Deng H. Z. (2010). Natural connectivity of complex networks. Chin. Phys. Lett. 27, 78902. doi: 10.1088/0256-307X/27/7/078902

[B56] Xia Y. Dong M. H. Yu L. Kong L. D. Seviour R. J. Kong Y. H. (2021). Compositional and functional profiling of the rhizosphere microbiomes of the invasive weed *Ageratina adenophora* and native plants. PeerJ 9, e10844. doi: 10.7717/peerj.10844 PMC7937340 33717679

[B57] Xiao H. F. Feng Y. L. Schaefer D. A. Yang X. D. (2014). Soil fungi rather than bacteria were modified by invasive plants, and that benefited invasive plant growth. Plant Soil 378, 253–264. doi: 10.1007/s11104-014-2040-x

[B58] Xiao Y. M. Wang J. Wang B. Fan B. Zhou G. Y. (2025). Soil microbial network complexity predicts soil multifunctionality better than soil microbial diversity during grassland-farmland-shrubland conversion on the Qinghai-Tibetan Plateau. Agric. Ecosyst. Environ. 379, 109356. doi: 10.1016/j.agee.2024.109356

[B59] Yang Y. Qiu K. Y. Xie Y. Z. Li X. C. Zhang S. Liu W. S. . (2023). Geographical, climatic, and soil factors control the altitudinal pattern of rhizosphere microbial diversity and its driving effect on root zone soil multifunctionality in mountain ecosystems. Sci. Total Environ. 904, 166932. doi: 10.1016/j.scitotenv.2023.166932 37690759

[B60] Yu F. K. Huang X. H. Duan C. Q. He S. Z. Zhang G. S. Liu C. . (2014). Impacts of *Ageratina adenophora* invasion on soil physical–chemical properties of *Eucalyptus* plantation and implications for constructing agro-forest ecosystem. Ecol. Eng. 64, 130–135. doi: 10.1016/j.ecoleng.2013.12.050

[B61] Yuan Y. Chen Z. Huang X. Wang F. Guo H. Y. Huang Z. . (2023). Comparative analysis of nitrogen content and its influence on actinorhizal nodule and rhizospheric microorganism diversity in three *Alnus* species. Front. Microbiol. 14, 1230170. doi: 10.3389/fmicb.2023.1230170 PMC10758417 38169791

[B62] Zhang G. L. Bai J. H. Tebbe C. C. Huang L. B. Jia J. Wang W. . (2021). *Spartina alterniflora* invasions reduce soil fungal diversity and simplify co-occurrence networks in a salt marsh ecosystem. Sci. Total Environ. 758, 143667. doi: 10.1016/j.scitotenv.2020.143667 33248759

[B63] Zhang D. J. Li J. J. Huang Y. M. Gao S. Zhang J. (2022). Root-soil facilitation in mixed *Eucalyptus grandis* plantations including nitrogen-fixing species. For. Ecol. Manag. 516, 120215. doi: 10.1016/j.foreco.2022.120215

[B64] Zhang M. M. Lu Y. Jin G. Z. Zhu B. (2025). Bacterial communities in litter are more sensitive to high nitrogen addition than fungal communities in a Korean pine (*Pinus koraiensis*) plantation. J. For. Res. 37, 23. doi: 10.1007/s11676-025-01961-5

[B65] Zhang M. Wang K. F. Shi C. Li X. Y. Qiu Z. L. Shi F. C. (2023). Responses of fungal assembly and co-occurrence network of rhizosphere soil to *Amaranthus palmeri* invasion in northern China. J. Fungi 9, 509. doi: 10.3390/jof9050509 PMC10219470 37233220

[B66] Zhao M. X. Lu X. F. Zhao H. X. Yang Y. F. Hale L. Gao Q. . (2019). *Ageratina adenophora* invasions are associated with microbially mediated differences in biogeochemical cycles. Sci. Total Environ. 677, 47–56. doi: 10.1016/j.scitotenv.2019.04.330 31051382

[B67] Zhao M. X. Yang Y. F. Zhang H. Li Q. Zhao X. X. Guo X. . (2024). Asymmetric succession in soil microbial communities enhances the competitive advantage of invasive alien plants. Microbiome 12, 265. doi: 10.1186/s40168-024-01989-5 PMC11662829 39707566

[B68] Zhou L. C. Huang L. J. Shi J. Z. Qin Y. D. Deng L. H. Liang S. Y. . (2026). Mixed plantations of *Alnus formosana* and *Pinus massoniana* improve ecosystem multifunctionality than monoculture plantations. Trees Forests People 24, 101171. doi: 10.1016/j.tfp.2026.101171

